# Ethical Considerations Related to Return of Results from Genomic Medicine Projects: The eMERGE Network (Phase III) Experience

**DOI:** 10.3390/jpm8010002

**Published:** 2018-01-03

**Authors:** Robyn Fossey, David Kochan, Erin Winkler, Joel E. Pacyna, Janet Olson, Stephen Thibodeau, John J. Connolly, Margaret Harr, Meckenzie A. Behr, Cynthia A. Prows, Beth Cobb, Melanie F. Myers, Nancy D. Leslie, Bahram Namjou-Khales, Hila Milo Rasouly, Julia Wynn, Alexander Fedotov, Wendy K. Chung, Ali Gharavi, Janet L. Williams, Lynn Pais, Ingrid Holm, Sharon Aufox, Maureen E. Smith, Aaron Scrol, Kathleen Leppig, Gail P. Jarvik, Georgia L. Wiesner, Rongling Li, Mary Stroud, Jordan W. Smoller, Richard R. Sharp, Iftikhar J. Kullo

**Affiliations:** 1 Department of Cardiovascular Diseases, Mayo Clinic, Rochester, MN 55905, USA; rlfossey@yahoo.com (R.F.); kochan.david@mayo.edu (D.K.); 2 Center for Individualized Medicine and Department of Medical Genomics, Mayo Clinic, Rochester, MN 55905, USA; winkler.Erin2@mayo.edu; 3 Department of Health Sciences Research, Biomedical Ethics Research Program, Mayo Clinic, Rochester, MN 55905, USA; pacyna.joel@mayo.edu (J.E.P.); olsonj@mayo.edu (J.O.); Sharp.Richard@mayo.edu (R.R.S.); 4 Department of Laboratory Medicine and Pathology, Mayo Clinic, Rochester, MN 55905, USA; sthibodeau@mayo.edu; 5 The Children’s Hospital of Philadelphia, Center for Applied Genomics, Philadelphia, PA 19104, USA; ConnollyJ1@email.chop.edu (J.J.C.); harrm@email.chop.edu (M.H.); behrm@email.chop.edu (M.A.B.); 6 Cincinnati Children’s Hospital Medical Center, Cincinnati, OH 45229, USA; Cindy.Prows@cchmc.org (C.A.P.); Beth.Cobb@cchmc.org (B.C.); Melanie.Myers@cchmc.org (M.F.M.); Nancy.Leslie@cchmc.org (N.D.L.); bahram.namjou@cchmc.org (B.N.-K.); 7 Department of Medicine, Division of Nephrology, Columbia University Medical Center, New York, NY 10027, USA; hm2673@cumc.columbia.edu (H.M.R.); ag2239@cumc.columbia.edu (A.G.); 8 Department of Pediatrics, Columbia University Medical Center, New York, NY 10032, USA; jw2500@cumc.columbia.edu; 9 Irving Institute for Clinical and Translational Research, Columbia University Medical Center, New York, NY 10032, USA; avf2117@cumc.columbia.edu; 10 Departments of Pediatrics and Medicine, Columbia University Medical Center, New York, NY 10032, USA; wkc15@cumc.columbia.edu; 11 Genomic Medicine Institute, Geisinger, Danville, PA 17822, USA; jlwilliams3@geisinger.edu; 12 Brigham and Women’s Hospital and Harvard Medical School, Boston, MA 02115, USA; lspais@bwh.harvard.edu; 13 Boston Children’s Hospital, Boston, MA 02115, USA; Ingrid.Holm@childrens.harvard.edu; 14 Center for Genetic Medicine, Northwestern University, Chicago, IL 60611, USA; s-aufox@northwestern.edu (S.A.); m-smith6@northwestern.edu (M.E.S.); 15 Kaiser Permanente, Seattle, WA 98101, USA; scrol.a@ghc.org (A.S.); leppig.k@ghc.org (K.L.); 16 Division of Medical Genetics, University of Washington, Seattle, WA 98195, USA; gjarvik@medicine.washington.edu; 17 Department of Medicine, Division of Genomic Medicine, Vanderbilt University Medical Center, Nashville, TN 37212, USA; georgia.wiesner@vanderbilt.edu; 18 National Human Genome Research Institute, Rockville, MD 20892, USA; lir2@mail.nih.gov; 19 Department of Medicine, Vanderbilt University Medical Center, Nashville, TN 37203, USA; mary.stroud@Vanderbilt.edu; 20 Center for Genomic Medicine, Massachusetts General Hospital, Boston, MA 02114, USA; jsmoller@partners.org

**Keywords:** Institutional Review Board, return of results, electronic health Record, informed consent, genome sequencing

## Abstract

We examined the Institutional Review Board (IRB) process at 9 academic institutions in the electronic Medical Records and Genomics (eMERGE) Network, for proposed electronic health record-based genomic medicine studies, to identify common questions and concerns. Sequencing of 109 disease related genes and genotyping of 14 actionable variants is being performed in ~28,100 participants from the 9 sites. Pathogenic/likely pathogenic variants in actionable genes are being returned to study participants. We examined each site’s research protocols, informed-consent materials, and interactions with IRB staff. Research staff at each site completed questionnaires regarding their IRB interactions. The time to prepare protocols for IRB submission, number of revisions and time to approval ranged from 10–261 days, 0–11, and 11–90 days, respectively. IRB recommendations related to the readability of informed consent materials, specifying the full range of potential risks, providing options for receiving limited results or withdrawal, sharing of information with family members, and establishing the mechanisms to answer participant questions. IRBs reviewing studies that involve the return of results from genomic sequencing have a diverse array of concerns, and anticipating these concerns can help investigators to more effectively engage IRBs.

## 1. Introduction

Studies that propose the disclosure of individual genetic results from research studies, as well as integration of these results into the electronic health record (EHR), are relatively novel protocols for Institutional Review Boards (IRBs) to review. Such studies employ designs that are necessary to begin translating genomics into clinical practice, but may introduce new kinds (or new scales) of risk [1,2,3]. The return of unexpected actionable genomic findings to those undergoing broad-scale genetic testing clinically and within research biobanks has proven to be controversial [4,5,6,7]. IRBs have varying degrees of experience with anticipating human subject vulnerabilities in such studies, and prior studies have demonstrated that IRBs differ in their interpretation and application of the same set of regulations [8].

The electronic MEdical Records and GEnomics (eMERGE) Network is a National Human Genome Research Institute funded consortium of research institutions across the United States, in which researchers with a wide array of experience and expertise in genomics, statistics, ethics, informatics, and clinical medicine recruit patients and leverage biorepositories linked to EHRs for genomic discovery and implementation studies [9,10,11,12]. The network, initiated in 2007, and now in its third phase, currently consists of 9 sites, two central sequencing facilities, and a coordinating center. Sites participating in this project include the Children’s Hospital of Philadelphia (CHOP), Cincinnati Children’s Hospital Medical Center (CCHMC), Columbia University, Geisinger, Partners HealthCare, Mayo Clinic, Northwestern University, Kaiser Permanente Washington, and Vanderbilt University Medical Center. The main focus of eMERGE phase III is genomic medicine implementation, including assessing the consequences of return of results (RoR) from genome sequencing to participants and their physicians through the EHR, and the social implications, ethics, and confidentiality issues that arise from such research [13].

We evaluated how different IRBs reviewed each eMERGE genomic medicine implementation study with the goal of identifying the common concerns and variation across sites. In sharing these experiences, our aim is to assist future investigators who may be preparing similar IRB protocols and spark a broader discussion of areas where consensus among IRB members may be lacking.

### 1.1. eMERGE III Site Study Descriptions

#### 1.1.1. The Children’s Hospital of Philadelphia (CHOP)

The RoR process involves two IRB protocols. The first, called ReLink, is based on re-contact of participants that are already enrolled in the biorepository at the Center for Applied Genomics (CAG) who had consented to participate in genomics research and to re-contact, but who had not previously been asked to consent to RoR (see Results and Table 1). Participants consented under ReLink, as well as new participants who have not previously enrolled in the CAG biorepository, have the option to consent to participate in CAG research as well as receive genomic test results, as part of a second protocol. Participants are recruited from the general pediatric population at CHOP, and the majority are less than 18 years of age. Results for conditions that have a pediatric age of onset, or which have screening recommendations prior to age 18 will be returned to all the participants: results for adult-onset conditions will be returned to individuals aged 18 years and over. All of the confirmed actionable variants will be returned by a genetic counselor with subsequent referral to a medical sub-specialist. Per protocol, a translator is required to be available for all consenting sessions.

#### 1.1.2. Cincinnati Children’s Hospital

The first protocol detailed processes for identifying and submitting biobank DNA samples from children with particular phenotypes. The protocol also includes prospective recruitment, enrollment, and obtaining samples from participants with phenotypes of interest. This protocol was not focused on RoR, but it allows for submitting a positive result to IRB as an unanticipated problem for review and approval before re-contacting the family. The second protocol is the primary RoR study created using focus groups which also contributed to the development of tools being used during the current eMERGE III RoR study. The preliminary focus groups and tool development work was possible through amendments to an existing protocol. The eMERGE III RoR study examines the prospectively enrolled adolescents’ and their parents’ independent and joint choices, and their responses to learning negative and positive results for their selected conditions from the eMERGE III sequencing panel. The second protocol indicates a specific aim to re-contact biobank participants and offer return of pathogenic/likely pathogenic (P/LP) results for 51 genes on the eMERGEseq panel.

#### 1.1.3. Columbia University

The goal of the Columbia eMERGE III project is to study the impact of genomic testing on the health of a diverse urban English and Spanish speaking adult patient population served by Columbia University Medical Center/New York Presbyterian Hospital system. Multiple recruitment methods were employed including recruitment in outpatient clinics, telephone recruitment, campus flyers, and community outreach. The study cohort includes 1500 prospectively recruited participants who were not selected for any medical condition. Confirmed P/LP variants in 70 selected genes and pharmacogenomic variants will be returned to the participant and placed in their EHR. A small subset of participants will be able to choose the results that they receive. Participants will have the option to meet with a genetic counselor and geneticist to review results. Longitudinal surveys will be used to analyze the effect of disclosing genomic sequencing results, including medical and non-medical utility, psychosocial impact, and sharing of information within families. The impact of genetic testing on clinical care is determined by periodic monitoring of EHRs.

#### 1.1.4. Geisinger

The Geisinger eMERGE III study involves a subset of participants that are enrolled in the systemwide biobank, MyCodeTM Community Health Initiative (MyCode CHI) who have been consented for re-contact regarding research opportunities and opted to receive research results. The objective of the MyCode CHI is to utilize genomic information to improve the ability to predict and prevent disease and optimize treatments for each individual participant. Return of results will focus on the return of P/LP variants in 76 selected genes (of which 60 are on the eMERGEseq panel). In 2500 individuals that were selected from MyCode CHI research exomes, including some that have been identified as having suspected P/LP variants, positive results are first reported to the primary healthcare providers of the participant. The laboratory report with the positive result is placed in the participant’s EHR. The primary healthcare provider has the option to contact the patient or may request that the Clinical Genomics Team contact the patient to return the result. Patients are given the option to meet with their primary healthcare provider, with the Clinical Genomics Team, or both. Health outcomes and patient satisfaction will be tracked by EHR review and patient survey.

#### 1.1.5. Mayo Clinic

The Mayo eMERGE III genomic medicine project is titled the Return of Actionable Variants Empiric (RAVE) study. Participants—3000 individuals, 2500 from Rochester, (mostly whites) and 500 from Phoenix, Arizona (Mexican Americans)—were identified from the Mayo Clinic Biobank and the Vascular Diseases Biorepository and chosen for having hyperlipidemia or colon polyps. The specific aims are to: (1) sequence samples using the eMERGEseq panel comprising 109 disease-related genes and assess the phenotypic correlates of rare putatively functional variants in study participants (2) return actionable results from 68 genes and 14 single-nucleotide variants (SNVs) to participants, and assess sharing of results with family members; (3) integrate genetic variants into EHRs for clinical care with linkage to clinical decision support; and, (4) assess health impact, cost effectiveness and ethical, legal and social implications of reporting genetic variants to participants and their families. In an ancillary study, the yield of cascade screening in families of participants with familial hypercholesterolemia will be examined.

#### 1.1.6. Northwestern University

Trained research coordinators and genetic counselors will prospectively recruit and consent 2355 Northwestern patients to receive both positive and negative results from the sequencing of 88 genes. These genes include the 56 American College of Medical Genetics and Genomics (ACMG) genes, 28 SNVs, and 7 pharmacogenomic variants, all of which were determined to have clinical utility. At the time of enrollment, participants will complete a brief survey assessing risk perceptions, early adopter status, understanding and potential use of genomic information, intent to share results, and thoughts about who should receive their results if they were to die before the testing was complete. Additionally, participants will be randomized to complete a brief survey to indicate their preference for receiving certain types of test results (e.g., results that are related to conditions where there is no treatment). Participants were also given the option to be surveyed and/or interviewed about the use and value of their results after receiving them. Negative and P/LP results will be placed in the EHR, making them available to all Northwestern physicians and to the participants through the Northwestern electronic patient portal (MyChart). Participants with negative results will be notified via mail and those participants with a previously known or unknown positive result will be notified via a phone call and/or in-person consultation with a genetic counselor and/or physician. Participants will be recruited from specialty clinics (e.g., Cardiology) and through advertising around the medical center. Additionally, 645 samples that were previously collected from participants in the eMERGE II Pharmacogenomics (PGx) study will undergo the same testing as the prospectively enrolled participants, but only positive results will be returned directly to them. These 645 PGx participants had previously consented to receive results, and those with positive results will be re-consented to allow for their results to be placed in their EHR.

#### 1.1.7. Partners HealthCare

The goal of the Partners HealthCare study is to study the penetrance and pleiotropic effects of rare variants derived from sequencing 109 genes in 2500 individuals that are enrolled in the Partners HealthCare Biobank and to conduct a randomized trial to assess the medical and economic outcomes of returning unanticipated genetic results to Biobank participants and their physicians. A subset of 2500 will undergo sequencing using the eMERGEseq panel. Return of results is focused on P/LP variants in ACMG 59 genes. For the majority of participants, sample collection was not through a fully Clinical Laboratory Improvement Amendments (CLIA) compliant process. When an actionable result is identified, the participant is notified by the Biobank by letter, email, and/or phone. Participants may opt out of receiving the result by completing an opt-out letter. For participants who opt in, the non-CLIA result is clinically confirmed. CLIA testing and all the subsequent follow-ups are conducted as part of clinical care. Testing is completed at the Partners Laboratory for Molecular Medicine (LMM) and a referral to a medical genetics sub-specialist is arranged. Participants are also given the option of signing a release to have the result communicated to their primary care physician (or other designated physician), with the additional option of medical genetics consultation. Under a separate IRB protocol, a randomized trial of return of results for P/LP variants in familial hypercholesterolemia genes (*LDLR*, *APOB*, *PCSK9*) will be conducted. Actionable variants in the three genes are identified from two sources: (1) genotyping using a genomewide array in up to 50,000 Biobank participants, and (2) sequencing using the eMERGEseq panel in 2500 Biobank participants. Individuals who choose to enroll will be randomized (1:1) to one of two arms: genetic result CLIA confirmed and disclosed immediately (Immediate Disclosure arm) or after one year (Delayed Disclosure arm) to the participant and their physician. Over the one-year trial, investigators will collect outcomes using participant surveys and EHR review to assess physician visits, laboratory testing, changes in medication prescriptions, LDL-C levels, medical costs, and the number of family members that are screened and treated as a result of intervention. 

#### 1.1.8. Kaiser Permanente Washington and University of Washington, Seattle

The genomic medicine study has three separate phases, each with a separate IRB application. eMERGE participants were drawn from a biorepository with broad consent for use in genomic research. Phase I of the IRB approval was submitted for approval on the submission of samples for sequencing. Phase II applies to the RoR process with permission to return results to patients with integration into the EHR. eMERGE III participants were enriched for a history of colorectal cancer, polyps, or Asian ancestry. Investigators will collect written informed consent to return results for the eMERGE III actionable gene list, including the 59 ACMG genes, to participants; both negative reports and variants of uncertain significance for the colon cancer or polyposis associated genes for those with colorectal cancer or polyps will be returned. All of the negative results are returned to the patient and their physician by mail. Clinically relevant results are returned to the patient in person by genetic counselor.

#### 1.1.9. Vanderbilt University Medical Center

The Vanderbilt eMERGE III site will build upon the PREDICT pharmacogenomic implementation program to develop an EHR pipeline that will deliver actionable variants from 89 genes and 20 pharmacogenomic variants to patients and providers, and to assess their response. 2500 adult participants who received care at Vanderbilt within the last three years were recruited from the PREDICT study as well as general adult clinics. Recruitment methods included; in person consent, phone consent, and electronic consent. P/LP and negative results from the gene and the pharmacogenomic variants will be uploaded to the EHR and will be disclosed to both the healthcare provider and the participant. This site will use a tiered disclosure system, where variants with modest clinical impact will be delivered by EHR message and/or letter and variants with high level of clinical impact will be communicated by phone or in person. The healthcare provider will be informed about the participant’s result, where they will disclose the results directly to their patient or request that study genetic counselors disclose the results. Negative results will be returned to participants by letter, and will be placed in the EHR. The genotype-phenotype relations will be studied by genome-wide association and advanced phenome-wide association methodology by expanding the known phenotyping library to better define the predictable clinical course or response to therapy. The investigators will also work with the other eMERGE sites to develop, implement, and assess tools to deliver new genomic information, measuring impact to ensure optimal benefit to patients.

## 2. Results

The time it took for sites to prepare for the initial IRB submission varied, based in part on the scope and scale of the corresponding projects and study personnel time assigned to protocol development. The average time taken to prepare for IRB submission was 100 days from the initiation of protocol development to IRB submission, with a median of 60 days. The number of IRB revisions among sites ranged from 11 (Mayo), to none (Partners’ return of eMERGEseq results to Biobank participants). Most sites did not report deferrals of review (a postponement of protocol review in order to make corrections or take action), however Mayo had two, and both Partners and the University of Washington each reported one. Cincinnati Children’s is the only site that reported having an IRB that had not previously been exposed to research protocols similar to the eMERGE III studies, however this IRB did have prior experience with projects involving genomic sequencing.

The time it took to obtain IRB approval ranged from 11–90 business days, with a mean of 39 business days and a median of 32 business days. At the time of publication, one site’s RoR protocol for an ancillary study, excluded from this statistic, had undergone two resubmissions and had not received IRB approval after seven months. The shortest approval period occurred at Partners; however, it was preceded by a multi-year process of discussions and meetings that included IRB leadership to develop, review, and iterate the return of results protocols and documents prior to submission. 

To recruit participants, seven sites utilized prospective recruitment for their studies (The Children’s Hospital of Philadelphia, Cincinnati Children’s, Columbia, Partners, Mayo-Rochester, Northwestern, and Vanderbilt), and four sites reported ongoing recruitment (Columbia, Partners, Mayo Arizona, and Vanderbilt). Mayo took five months to complete participant enrollment at the Rochester site, and Washington took two months. Four sites required approval from an ancillary committee (CHOP, Geisinger, Partners, and Mayo) prior to the IRB review of protocol changes. CHOP required approval from the Clinical Decision Support group for protocol changes, and at Mayo, approvals from the pediatric and adolescent research and Clinical and Translational Science Committee were required for protocol review. 

### 2.1. Common IRB Recommendations

Common issues raised by IRBs included: (a) attempting to strike a balance between the degree of detail and readability needed for informed consent, (b) the process for determining actionable genes in pediatric populations, (c) the specific language detailing the full range of potential risks, (d) options for participants to receive specific categories of results, (e) considerations for withdrawal, and (f) availability of research specialists to answer participant questions. Each of these issues is described in detail below.

#### 2.1.1. Identifying the Appropriate Balance of Content Volume, Degree of Detail, and the Readability Needed for Informed Consent

Investigators and IRBs noted that it was important to prepare the content of consent forms so that the participants could read the consent form in its entirety, ask questions, and to ensure that the language in the consent is understandable. Indeed, several IRBs commented that the language in the informed consent needed to be easy to read and comprehend. Cumulatively, the mean Flesch-Kincaid grade level of site consent forms from all sites was 9.7—higher than the recommended grade level of 8 for informed consent [14]. Child assent forms at pediatric sites ranked higher in readability, with a mean Flesch-Kincaid grade level of 8.2. The Flesch-Kincaid grade levels of consent forms ranged from 7.8 to 12.1 (CHOP and Columbia respectively).

Interestingly, Mayo’s IRB recommended that the consent form wording be modified to specify the type of genomic screening being performed and to include a description of the sequencing in easily understandable terms. Columbia included institutionally standardized text regarding genetics, information about the Genetic Information Nondiscrimination Act (GINA) [15], and the types of results that may be returned for genomic studies into their consent form. Northwestern’s IRB requires a paragraph describing GINA for any genomic study. Consent forms from all of the sites except one directly referenced GINA in their consent forms. The remaining site’s consent forms discussed the healthcare protections that are provided by GINA without directly referencing the act. 

Partners’ IRB recommended setting expectations regarding the low likelihood of receiving positive genetic test results, mentioning that the results are not derived from clinical tests (since the site’s samples were not collected under CLIA regulations, and that terminology regarding “actionable results” be modified as follows: “It is important to remember that research results are not always meaningful and are not the same as clinical tests. While you should not expect to receive any results from your participation in this research, if experts from the Biobank decide that research results from your sample are of high medical importance, we will attempt to contact you”.

IRBs reiterated that if non-English speaking individuals were being consented, additional considerations must be provided to ensure that potential participants are fully aware of its content. Columbia, Mayo, and Northwestern offered consent forms, supporting literature, and media in both English and Spanish languages. Mayo’s Arizona site is exploring the option of having test reports translated into Spanish.

Several sites reported differences between investigators and their respective IRBs regarding the amount of information and the level of detail individuals should receive in order to participate. For instance, Mayo’s IRB recommended revision of the main consent form to include mention of the specific type of genomic screening (i.e., whole exome, whole genome sequencing) as well as a description of the sequencing in lay terms. Other IRBs however, did not determine that this level of detail was essential to include in the informed consent document. Vanderbilt offered in-person, over the phone, and electronic consent options for the patients. Columbia reported having an IRB designated specifically for genomic research studies, which was perceived to be beneficial for genomic protocol review and facilitating study team communications with the IRB. 

#### 2.1.2. The Process for Determining Actionable Genes in Pediatric Populations

To address additional concerns of returning actionable genes in pediatric populations, the ACMG and American Academy of Pediatrics have provided guidance for the return of incidental findings to children [16,17]. In addition to these guidelines, the CHOP and CCHMC eMERGE III sites have instituted the following procedures to determine the actionability of genomic findings. At CHOP, results for conditions that have a pediatric age of onset, or which have screening recommendations prior to age 18 years will be returned to all participants, and results for adult-onset conditions will be returned to individuals aged 18 years and over. CCHMC used an internal group of genetics and ethics experts to determine actionable genes relevant to the prospective adolescent/parent and the biobank study populations. The list for CCHMC’s prospective adolescent/parent cohort included the ACMG 56 genes, as well as additional eMERGE selected genes that had known associations with disorders that could potentially manifest in childhood, such as *CFTR*, or genes for conditions that CCHMC investigators had a particular interest in, such as *BMPR2*. Genes for autosomal recessive disorders that were not expected to be found in a healthy pediatric population, such as *TCIGR1* for osteopetrosis, were in the initial IRB approved list to allow the cohort to make choices about learning carrier status for relevant eMERGEseq genes. Any additional genes later designated as actionable by the eMERGE Clinical Annotations workgroup were discussed with an internal genetics expert who was also a co-chair of the IRB, and then submitted as an amendment to the protocol. 

The genes approved by the internal expert group for the larger biobank cohort included the ACMG 56 minus the adult onset cancer predisposition genes. Two genes associated with potential polyposis syndromes (*SMAD4*, *BMPR1A*) and *KCNJ2*, associated with arrhythmia phenotypes, were included in the list of genes considered actionable among pediatric populations.

#### 2.1.3. Specific Language Detailing the Full Range of Potential Risks 

Each site’s consent form incorporated similar language to communicate potential risks (Table 2). Specific risks included possible loss of privacy, discrimination for life, long-term care, and disability insurance, health insurance discrimination from small employers, discovery of non-genetic family relationships, unknown psychological impact of results, as well as the possible complications of a blood draw. At Partners, the IRB expressed concern that current federal protections for pre-existing conditions may have an uncertain future, and that placement of genetic test results in the EHR could lead to a diagnosis that is considered a pre-existing condition. Partners’ IRB recommended including language describing this risk in the informed consent form.

#### 2.1.4. Options for Participants to Receive only Specific Categories of Results 

At the time of consent, the participants at four sites were given the option to choose the types of results they would like to receive. At Mayo, the choices included the option to receive only primary results (results that are linked to the indication for testing), the option to receive all test results regardless of indication (primary and secondary), or the option to receive primary results and medically actionable secondary results. At Columbia, a small subset of the study population (<400 participants) were offered a choice regarding the types of results that they would like to receive. Options included conditions with effective treatments available, and conditions with less effective treatments available. Participants were further allowed to choose which conditions they would or would not like to receive results for. Northwestern randomized the first 1564 participants into groups that either receive all results, or choose to receive results for specific types of results. Participants chose whether to receive results for the following categories: conditions where treatment is available for symptoms, conditions where treatment is not available for symptoms, results related to dementia or a risk to develop dementia, results related to cancer or a risk to develop cancer, results with implications for learning and behavior, results reflecting the participant being a carrier for a disease, and results where researchers are still investigating a link between the gene and disease. CCHMC’s adolescent protocol promoted choices between preventable and non-preventable conditions, treatable and non-treatable conditions, and whether to receive carrier status results, and the results for adult onset conditions.

At Partners, participants (other than those enrolled in an ancillary randomized controlled trial returning results that are related to familial hypercholesterolemia) can opt-out of receiving specific results or undergoing clinical confirmation after being notified that an actionable result has been found.

#### 2.1.5. Options for Withdrawal

Each site was asked to clearly define at what point in the study a participant could choose not to receive results. At most of the sites, the IRBs opined that if investigators had not placed results in the EHR, the participant could withdraw from the study at any time. Notably, it was emphasized that results could not be removed from a participant’s clinical record after EHR entry had taken place. It was determined that results would be placed in the EHR following the disclosure of genetic test findings in a clinical encounter. 

#### 2.1.6. Availability of Study Team Members to Answer Participant Questions 

IRBs reiterated that it was important for a study team member to be available to answer any questions that participants may have about the study. A phone number and or email would be provided so that participants could ask questions that may arise at a later time. For sites returning negative test results, additional considerations for the study team regarding how to prepare and deliver negative results were introduced. IRBs requested that genetic counseling be readily available for participants with questions regarding their genomic findings, positive or negative.

### 2.2. Disclosure of Results

Genetic counselors were preferred for genomic RoR at most sites. IRBs stressed the importance of enabling participant access to genetic counseling services. Seven sites will return positive results to participants during an in-person genetic counselor appointment or over the phone. Positive results are defined as P/LP variants in one or more of the actionable genes on the eMERGEseq panel or actionable genotyping results from the 14 SNV panel. For research purposes, some of the sites opted to include pharmacogenetic variants for return. At Columbia, the IRB did not explicitly ask that investigators differentiate between actionable variants, positive results related to indications, incidental findings, and pharmacogenetic results, and left much of this at the discretion of study investigators. Cincinnati Children’s investigators opted to have a genetic counselor to return test results via phone call or through an online patient portal, which was agreeable to their IRB. At two sites, a health care provider will disclose results, initially followed by in-person genetic counseling, if requested. Some sites will include positive results in a letter sent to study participants. Five sites intend to disclose negative results to study participants via phone, letter, or an online patient portal. At sites returning negative test results, IRBs requested that those receiving negative results should be encouraged to contact a genetic counselor if they have questions.

IRBs at four sites recommended providing a gene list to participants following the disclosure of results. Partners provides a complete gene list to study participants via a participant accessible website. At Northwestern, participants are provided access to a patient-friendly gene list. At Mayo, the study team expressed concerns that the contents of a gene list may be difficult for many participants to understand. The consensus was that after a genetic counseling or genetic disclosure session occurs, each participant will be given a letter explaining their positive results in an easily understandable manner. 

In the event that study participants could not be contacted regarding actionable results, varying approaches were proposed at the eMERGE sites. At some sites, primary care providers would be contacted after a predetermined period of time (e.g., six months) and the results placed in the EHR. At another site, if multiple attempts to reach participants were unsuccessful, participants were considered lost to follow-up.

### 2.3. Return of Negative Results

At the five eMERGE III sites returning negative results, four sites proposed to return negative results by letter with the option of speaking to a genetic counselor by phone if participants had questions. Cincinnati Children’s randomized the return of negative results by a phone call or through a patient portal. At Mayo, within the first deferral, the IRB requested that the study team consider “using an alternative method of communicating, such as in-person, for both the actionable and the negative genetic results with participants. Communication should be done in a manner that minimizes undue risks that are related to the learning of genetic results. An in-person communication would allow for the participants to have their questions/concerns related to results immediately addressed”.

In response to this request the study team accepted that a null result from genetic testing may not indicate a lower risk for disease, and that negative results may still be of interest to study participants. It was also reiterated by the study team that a genetic counselor would be available for any participant receiving negative results who requested more information about their result. The IRB accepted this response and approved the return of negative results via a letter with the option to speak with a genetic counselor on the phone if questions arose.

### 2.4. Sharing of Results with Family Members

Some IRBs raised concerns about how participants would be counseled regarding the sharing of information with family members who might be impacted by the genetic findings. At one site, investigators proposed, in addition to genetic counseling, to direct participants to the eMERGE MyResults.org website, which describes implications for family members. Most sites did not include direct contact of family members in their research protocols and were not asked to do so by their IRBs. A few sites reported evaluating the inclusion of family communication, or screening strategies that were specific into their studies. Geisinger included the study of family communication as one of their aims and are evaluating through focus groups and qualitative interviews, participant preferences for family sharing of genomic information. Kaiser Permanente of Washington received supplemental funding to identify potential family members who are also patients within the healthcare system. Mayo received supplemental funding to perform cascade screening on family members of probands who had results indicating familial hypercholesterolemia. The investigators sought for and obtained IRB approval to directly contact family members of participants with positive genetic test results for familial hypercholesterolemia to facilitate cascade testing. Participants are given the option of contacting first degree relatives themselves or allowing the study team to send pre-prepared letters to their relatives explaining their risk of having familial hypercholesterolemia and asking them if they are interested in participating in the study. This will allow the site to determine whether family members who are contacted elect for genetic testing and/or make changes based on newly discovered information.

## 3. Discussion

The goal of this paper was to provide a summary of the genomic medicine projects in eMERGE III and the IRB responses to the proposed RoR projects at each site. A unique aspect of these studies is that they straddle the boundary between research and clinical implementation. Such studies involving the return of genomic research findings are relatively novel, and are expected to become more common in the future [18]. The genomic medicine studies across the network are conceptually similar, but vary from site to site. All IRBs had previously reviewed projects involving genomic sequencing. Understandably, IRBs emphasized that participants are provided with information relevant to the study during consent, understand the implications that genomic information may have for their care, and that the participants have readily available access to genetic counseling and/or study team personnel for questions. The most common IRB comments that are related to balancing the detail and readability of informed consent materials, differentiating actionable genes in adult and pediatric populations, specifying the full range of potential risks to participants, providing options for participants who may want to receive limited results or withdraw from the study at a later stage, counseling regarding the sharing of information with family members, and having in place mechanisms to answer participant questions.

The time to prepare eMERGE protocols for IRB submission, the number of IRB revisions across sites, and the time from submission to approval varied from site to site. Overall, communicating with IRB staff prior to and during protocol review appeared to be beneficial for clarifying study considerations that were specific to genomic RoR studies and may facilitate the process and reduce the time to approval. In addition to providing supplemental explanations to an IRB regarding genomic aspects of a study, including institutionally standardized literature and study materials, may promote participant informed consent [19], and help to facilitate the review process. Concerns related to participant understanding of study results were raised by multiple site IRBs. To mitigate IRB concerns, investigators should plan to provide for robust participant support such as supplementary materials during consent, ensuring that genetic counseling or appropriate educational support is available to participants at the time of disclosure and following, and providing easily understandable interpretations of testing result. 

With the current possibility of repeal of the Affordable Care Act, participant concerns about access to care with pre-existing conditions, and therefore the placement of genetic data in the EHR, is likely to increase. In consent forms, all of the sites included text mentioning the protections afforded by GINA [15]. Sites were not required to include certificates of confidentiality in their consent forms. A recently introduced policy will essentially apply the protections of a certificate of confidentiality to NIH-funded investigations without the investigator having to apply for one [20]. 

Integrating the return of genomic research findings into clinical practice carries considerable implications for all study participants [10]. It is critical that the participant understands the consent form, is educated about their role in the research study, and that any questions raised by the participant are answered by a member of the study team. Concerns regarding participants’ understanding of consent form contents have been documented [21,22]. Issues may arise when obtaining consent from individuals with low literacy, language barriers, religious beliefs, false expectations, as well as being in vulnerable populations and children [23].

Genomic studies enrolling children have additional regulatory requirements regarding child assent and parent or guardian permission, and unique considerations for the return of genomic research findings [16,17]. A child must understand that he/she is not obligated to participate [24], and that neither his/her medical team nor his/her parents will be upset if they choose not to participate. Disclosing results to pediatric participants consistent with predictive genetic testing for adult onset conditions has ethical implications, and could place undue burden on pediatric participants. The ACMG and American Academy of Pediatrics have recommended that testing children for adult-onset conditions should not be performed until a child has reached maturity. If genetic testing is performed and the results secondary to the indication for genetic testing are identified, disclosure is recommended if there is either preventative value to the child, or diagnostic value to undiagnosed parent(s) for adult onset conditions [16,17].

Many of the genes tested in the eMERGEseq panel represent relatively prevalent autosomal dominant disorders, raising the possibility of family (cascade) screening. When implemented effectively, cascade screening can enable awareness and preventive care for families, reducing the risk of adverse medical events [25,26]. Cascade screening for familial hypercholesterolemia, for instance, has proven to be highly effective at identifying affected individuals and improving disease management when implemented in The Netherlands [27,28,29], and has been recommended by the CDC for use in the United States (US) [30]. Cascade screening has numerous implications for the proband and potentially affected family members. However, privacy concerns forbid the direct contact of family members by health care providers. At Mayo, investigators obtained approval to contact first degree relatives of participants with familial hypercholesterolemia, with participant consent.

It is universally accepted that research participants have the right to withdraw from a study at any time [31,32], and IRBs stressed this in their communications with study teams. However, the translation of genomic research results from research to clinical use introduces a situation in which it may not be possible to remove clinical results from their EHR, complicating the participant’s right to withdraw from the study. Once a participant’s genetic counseling session has begun, and results are disclosed, the transition from a research finding to a clinical report occurs and participants may not be able to withdraw their information from the EHR regardless of their study enrollment status. 

Despite the novel nature of the genomic medicine studies proposed by eMERGE sites, only one site encountered substantial hurdles while obtaining IRB approval. At this site, as part of an ancillary study, the investigators proposed to delay the RoR for a subset of participants in a randomized trial. The IRB requested that a licensed physician investigator or genetic counselor obtain consent for the study and raised ethical concerns about delaying the RoR longer than is necessary. Concerns regarding how to handle the return of non-CLIA research results and to what extent the results can be communicated to participants prior to clinical confirmation with a separate sample were also raised.

Although this study was carried out in the US, many of the underlying principles of biobanking, the return of genomic results, and study protocol review are applicable worldwide. International investigators should refer to their local legislation and carefully consider protections for study participants when considering projects of a similar nature. Stringent quality control during sequencing, confirmation of sequencing findings, and the characterization of variants should be considered by study teams conducting genomic medicine implementation studies.

## 4. Materials and Methods 

We reviewed the aims of the genomic medicine projects at each site and compared informed consent documents for each project. The eMERGEseq panel comprises 109 disease-associated genes, of which 68 genes were deemed actionable by the eMERGE investigators [10]. Once P/LP variants are identified these are to be returned to participants, usually by a genetic counselor.

Each site completed a questionnaire related to the IRB review of their protocol including: the time it took to prepare for cohort selection and IRB submission, whether ancillary committees were involved in the review process, the total number of protocol revisions, how many deferrals and review withdrawals occurred, and the time it took for the study to receive IRB approval. Surveys were administered retrospectively to participating eMERGE sites. IRB submission time was estimated from the start-date of protocol development, up to submission. Questionnaires and consent forms were received from each eMERGE III site and were reviewed independently by two authors. Common themes were identified. The Flesch-Kincaid [33] readability of site consent forms was calculated using Microsoft Word 2010. Questionnaires instructed site investigators to describe IRB interactions that were related to specific topics, such as consent forms, genetic counseling, study withdrawal, RoR choices for participants, the placement of study results into the EHR, contacting family members of study participants, intent for negative results, and any other issues raised by the IRB. Common concerns reported by investigators at each site, and the corrective actions that were made by the study team to remediate them, were assessed.

## 5. Conclusions

We summarized the genomic medicine projects in eMERGE III to provide contextual background related to the logistical aspects of the IRB review at each site. Numerous conceptual issues were raised by IRBs, including those that were related to incidental findings, informed consent, placement of results in the EHR and contacting family members for screening. However, there were no substantial concerns which prevented IRB approval of these translational genomic research projects. Common IRB recommendations included using language that is easily understandable, addressing special considerations for return in pediatric populations, including specific language communicating possible risks to study participants, establishing mechanisms to answer questions regarding any portion of the research, and ensuring that participants understand their options for withdrawal from the study (Table 3). Communicating with IRB staff during protocol review and including supplemental explanations regarding genomic aspects of the studies may facilitate the review process. Consent forms for genomic RoR studies bear the burden of introducing potentially complex concepts to participants. The continued refinement of consent form language for genomic studies and the creation of specific IRB review committees that are familiar with genomic implementation studies may prove beneficial to investigators and sites pursuing large scale genomic research studies with the potential for returning results. The findings from our study may be informative for investigators who plan to conduct studies returning genomic sequencing results within the context of biobanks. The findings of this study may also be helpful to IRBs at research institutions which are likely to increasingly review such projects. 

## Figures and Tables

**Table 1:** Summary of each electronic MEdical Records and GEnomics (eMERGE) site’s genomic medicine study.

Institution	Study Population	Enrollment Method	Enrollment Qualification	Number of Genes for RoR	Returned Results	Return Mechanism	Measurements & Follow-up
The Children’s Hospital of Philadelphia	3000	Retrospective Prospective	Primarily pediatric participants	Up to 68 for adults, ~59 pediatric	Positive results only. Adult-onset not returned to participants <18 years old	Positive results returned by Genetic Counselor	Surveys Electronic Health Record review
Cincinnati Children’s Hospital	3000	Retrospective (2800)	Pediatric biobank samples with phenotypes of interest	51	ACMG 56 genes without adult onset, positive results only	Positive results returned by phone (Genetic Counselor)	Survey Electronic Health Record review
		Prospective (200 dyads)	Adolescents capable of making decisions and one of their parents	86 possible	Adolescent-Parent joint selection of conditions informed by 86 genes	Negative results randomized to patient portal or telephone (Genetic Counselor)	Surveys Electronic Health Record review
Columbia	2500	Prospective (1500)	Adults	70	Positive and negative results will be returned	Actionable positive results returned by Genetic Counselor	Surveys Electronic Health Record review
		Retrospective (1000)				Negative results via letter All results placed in the Electronic Health Record	
Geisinger	2500	Retrospective	Individuals selected from MyCode CHI research exomes	76	P/LP variants in 76 genes are returned	Primary care provider notification Electronic Health Record entry Positive results returned by Primary care provider and/or Clinical Genomics Team	Survey Electronic Health Record review
Mayo	2500 Rochester, MN	Retrospective	Hyperlipidemia and Colon Polyps	68	Patient elects for either: Primary, Primary + actionable secondary, or Primary + secondary	Genetic Counselor, letter sent to those with negative results	Cascade screening Psychosocial domains Clinical outcomes
	500 Phoenix, AZ	Prospective					
Northwestern	3000	Prospective (2355)	Adult patient at Northwestern Medicine (NM)	88	Clinically relevant findings	Genetic Counselor and/or Physician Negative results in mail	Surveys Interview Electronic Health Record review
		Retrospe ctive (645)					
Partners	2500	Retrospective	Adult Biobank participants who have agreed to be re-contacted	59	P/LP variants in ACMG 59 genes	Genetic counselor notifies of actionable result (Non-CLIA) If opt in, CLIA sample obtained for clinical confirmation	Surveys Electronic Health Record review
	~100	Prospective	Adult Biobank participants who have agreed to be re-contacted	3	P/LP variants for Familial Hypercholesterolemia Immediate disclosure (<1 month) vs. delayed disclosure (1 year)	Electronic Health Record entry; physician notification; result mailed to participant	
Kaiser Permanente Washington & University of Washington	2500	Biobank and Retrospective	Colon Cancer and Polyposis with Asian Ancestry	68	Clinically relevant findings	Genetic Counselor and/or Physician Negative results in mail	Surveys, family cascade testing and communication tools, Cost assessments, Electronic Health Record review for referrals
Vanderbilt	2500	Prospective	Adults	109	Actionable results	Positive results returned to the patient and Vanderbilt primary care provider Negative results letter sent to Electronic Health Record for entry	Survey Electronic Health Record review

ACMG: American College of Medical Genetics and Genomics, P/LP: Pathogenic or likely pathogenic.

**Table 2:** A summary of the content of consent forms from each eMERGE site.

Institution	Option to Withdraw	Benefits	Pediatric Patients?	Risks	Certificate of Confidentiality	Study Duration	Secondary Findings
The Children’s Hospital of Philadelphia	Any time, shared data cannot be revoked	Results may allow for early treatment, scientific knowledge	Yes	Loss of privacy, stigmatization, insurance discrimination (life, disability, long-term care)	Yes	Indefinite	No
Cincinnati Children’s (Prospective cohort)	Any time up to the end of study	Up to $50 (Study visit + 2 surveys) Results may allow for early treatment, scientific knowledge	Yes, ≥13 Years of Age	Errors in testing (False + or −), false sense of wellness, anxiety, distress, insurance discrimination (non-health)	No	Up to 2 years	All potential results incidental and optional at consent
Columbia	Any time before the data is in the EHR, shared data cannot be revoked	Identification of important health information and up to a total of $75 gift cards for completing up to three surveys	No	Anxiety, depression, sadness, guilt, discrimination (other institutions, life, disability, long-term care insurance) Loss of privacy	Yes	Indefinite	All actionable results will be returned
Geisinger	Any time, shared data cannot be revoked	Identification of important health information	No	Loss of privacy, insurance discrimination (life, disability, long-term care)	Yes	Indefinite	Yes if treatable
Mayo	Any time up to EHR entry, shared data cannot be revoked	No-cost genetic screen, value to family, scientific contribution, Identification of important health information	No, possible enrollment of children for cascade screening	Anxiety, Cost of additional care, loss of privacy, discrimination (non-health insurance or <15 employees)	No	Indefinite	Optional at Consent
Northwestern	Any time up to EHR entry. Shared data cannot be revoked	$50 if selected for interview, scientific contribution, Identification of important health information	No	Discrimination (non-health insurance), loss of privacy, discovery of non-genetic siblings/family members, unknown psychological impact of results	No	3 Years	Yes
Partners	Any time, Results in the EHR cannot be removed	Identification of potentially relevant health information to individual and family	No	Loss of privacy, loss of confidentiality with potential for insurance or employment discrimination	No	Indefinite (Biobank)	Yes
						13 months (Ancillary Clinical Trial)	
Kaiser Permanente Washington & University of Washington	Any time, EHR information cannot be removed	Knowledge of medical conditions	No	Loss of confidentiality, emotional distress/discomfort, may affect reproductive decisions, genetic discrimination (non-health insurance, employers <15 employees)	No	Subject involvement ends at the return of results, Research use ends later	Yes
Vanderbilt	Any time, Shared data cannot be revoked	$45 ($25 gift card for first survey, $10 for remaining two)	No. 21+ years	Loss of privacy with DNA sample	No	6+ years for research record	Yes

**Table 3:** Summaries of Institutional Review Board (IRB) recommendations.

**Recommendations from IRBs at ≥2 Sites**
Language in consent documents must be easily readable and understandable for all
Materials must be provided to ensure that individuals with low English literacy can understand study information prior to consent
Investigators must define the point in time for participants that study findings can be withdrawn, and clarify that following results disclosure, results cannot be removed from a participant’s health record
Mention protections afforded by GINA, potential impact on long-term care and disability insurance
Determine which genes are associated with medically actionable conditions in pediatric populations, and which are specific to adult onset conditions
Include loss of privacy and potential insurance discrimination as risks
Consider counseling participants regarding the sharing of genetic test results with family members
Consider providing a gene list to the study participants
**Specific IRB Recommendations (Limited to IRBs at 1 Site)**
Consider mentioning that current federal protections for pre-existing conditions may have an uncertain future—Returned results may be considered to be pre-existing conditions
Consider describing the low likelihood of receiving positive test results
Explain that results from samples not obtained under CLIA are considered research findings and may not be as meaningful as clinical tests
Consider educational resources for participants and their healthcare providers that describe in greater detail the proposed genome sequencing techniques.
Consider using an alternative method of communicating, such as in-person, for both the actionable and the negative genetic results with participants
